# The effect of pericolic lymph nodes metastasis beyond 10 cm proximal to the tumor on patients with rectal cancer

**DOI:** 10.1186/s12885-020-07037-3

**Published:** 2020-06-19

**Authors:** Xuyang Yang, Erliang Zheng, Lina Ye, Chaoyang Gu, Tao Hu, Dan Jiang, Du He, Bing Wu, Qinbing Wu, Tinghan Yang, Mingtian Wei, Wenjian Meng, Xiangbing Deng, Ziqiang Wang, Zongguang Zhou

**Affiliations:** 1 Department of Gastrointestinal Surgery, West China Hospital, Sichuan University, No. 37 Guo Xue Alley, Chengdu, 610041 Sichuan Province China; 2 Department of General Surgery, Xi’an Central Hospital, Xi’an, China; 3 Department of Pathology, West China Hospital, Sichuan University, No. 37 Guo Xue Alley, Chengdu, 610041 Sichuan Province China; 4 Department of Radiology, West China Hospital, Sichuan University, No. 37 Guo Xue Alley, Chengdu, 610041 Sichuan Province China

**Keywords:** Rectal cancer, Paracolic lymph node, Proximal resection margin, Risk factor, Prognosis

## Abstract

**Background:**

This study aims to determine the real incidence of pericolic lymph nodes metastasis beyond 10 cm proximal to the tumor (pPCN) and its prognostic significance in rectal cancer patients.

**Methods:**

Consecutive patients with rectal cancer underwent curative resection between 2015 and 2017 were included. Margin distance was marked and measured in vivo and lymph nodes were harvested on fresh specimens. Clinicopathological characteristics and oncological outcomes (3-year overall survival (OS) and disease-free survival (DFS)) were analyzed between patients with pPCN and patients without pPCN (nPCN).

**Results:**

There were 298 patients in the nPCN group and 14 patients (4.5%) in pPCN group. Baseline characteristics were balanced except more patients received preoperative or postoperative chemoradiotherapy in pPCN group. Preoperative more advanced cTNM stage (log-rank *p* = 0.005) and intraoperative more pericolic lymph nodes beyond 10 cm proximal to the tumor (PCNs) (log-rank *p* = 0.002) were independent risk factors for pPCN. The maximum short-axis diameter of mesenteric lymph nodes ≥8 mm was also contributed to predicting the pPCN. pPCN was an independent prognostic indicator and associated with worse 3-year OS (66% vs 91%, Cox *p* = 0.033) and DFS (58% vs 92%, Cox *p* = 0.012).

**Conclusion:**

The incidence of pPCN was higher than expected. Patients with high-risk factors (cTNM stage III or more PCNs) might get benefits from an extended proximal bowel resection to avoid residual positive PCNs.

## Background

Radical surgery remains the mainstay of treatment for rectal cancer. The primary goal of curative resection includes en bloc removal of the tumor with adequate resection margins and complete removal of regional lymph nodes. Whereas a distal resection margin of ≥1 cm is well accepted, the optimal extent of proximal resection is still unclear [1]. In 1954, Grinnell RS found that rectal tumor proximal intramural spread was present within 5 cm [2]. After that, the 5-cm rule of proximal bowel resection margin was adopted in surgery [3]. However, several studies had challenged the 5-cm rule for better oncological outcomes achieved with extended proximal bowel resection [4–6]. Compared with tumor intramural spread, the presence of pericolic lymph nodes metastasis imposed an additional requirement for bowel resection [7]. Upward spread is the main course of lymphatic spread in rectal cancer, yet the status of pericolic lymph nodes especially those located beyond 10 cm from the tumor proximal margin is not well defined. Previous studies suggested that pericolic lymph nodes metastasis beyond 10 cm from the primary tumor was rare (0–1.8%) [8–11]. Based on careful pathological studies, Japanese guidelines also recommended the proximal resection margin of 10 cm in rectal cancer, and this rule was widely adopted in eastern countries [12].

In clinical practice, however, we had noticed several rectal cancer patients with pericolic lymph nodes metastasis beyond 10 cm proximal to the tumor (pPCN). We speculated the real incidence of pPCN might be underestimated in previous studies due to the tissue shrinkage after removal from in vivo or fixing with formalin. Therefore, since 2015, we attempted to perform an observational study to harvest these regional lymph nodes on the fresh specimen with margin distance measured prior to bowel resection and to analyze the definite incidence of pPCN and its impact on prognosis.

## Methods

### Patients

**Fig. 1:**
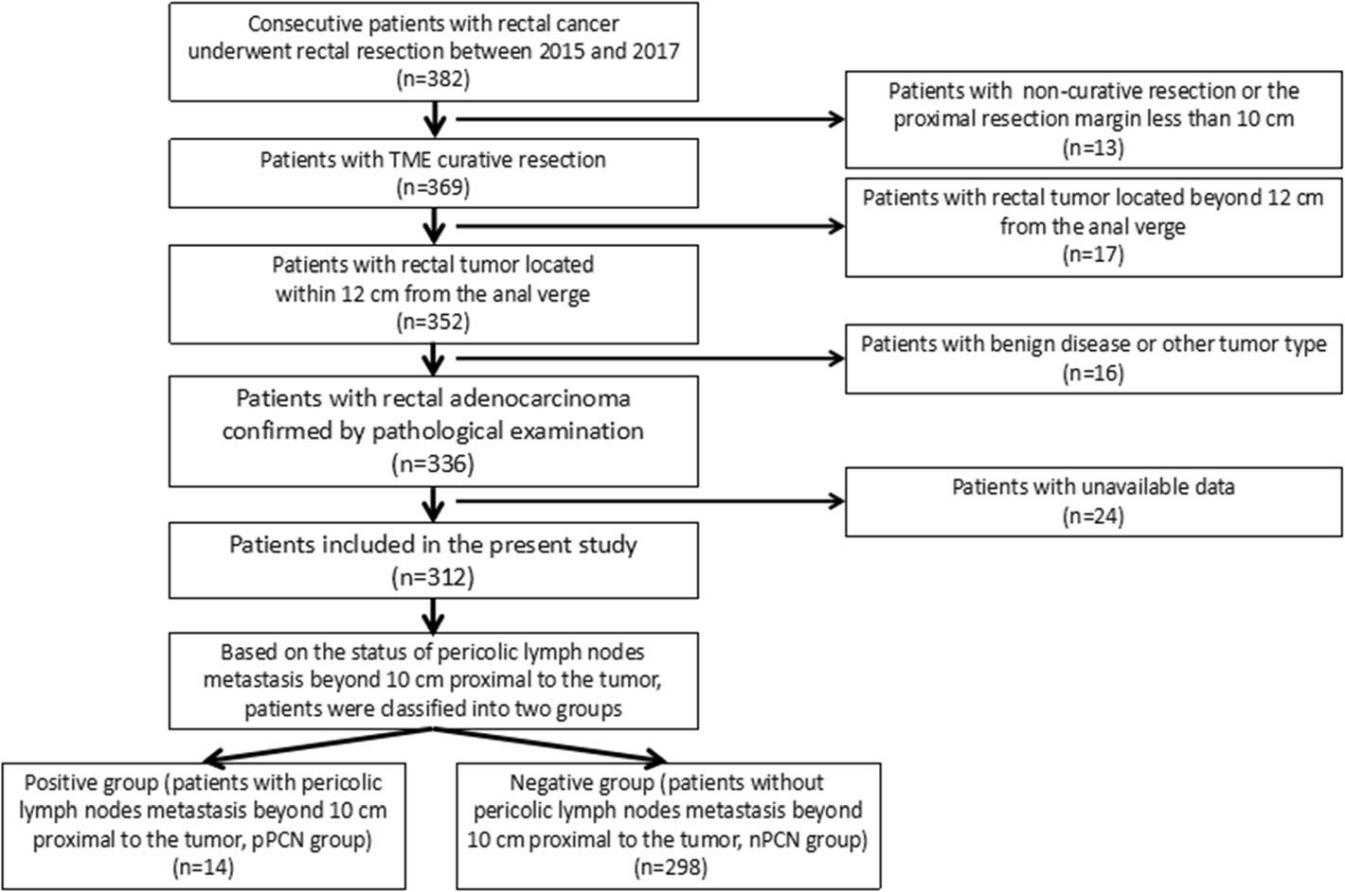
The research process flow chart

Between January 2015 to May 2017, consecutive patients with rectal cancer underwent radical resection in our hospital were included. All data were collected from the prospective database. The inclusion criteria were as followed: rectal adenocarcinoma confirmed by pathology; tumor located within 12 cm from the anal verge; patients underwent radical resection; the proximal resection margin more than 10 cm; IV stage patients with potentially resectable metastatic lesions. Patients with synchronous colorectal cancer, palliative resection, the proximal resection margin less than 10 cm, or missing data were excluded. In this study, patients with pericolic lymph nodes metastasis beyond 10 cm proximal to the tumor were designated as the patients within pPCN group, while those without pericolic lymph nodes metastasis beyond 10 cm proximal to the tumor were designated as patients within nPCN group. Approval from the Ethics Committee of our hospital and informed consent from all patients before the operation were obtained. The research process flow chart was shown in Fig. 1.

### Preoperative staging and treatment strategy

The preoperative clinical evaluation included physical examination, laboratory tests, colonoscopy, endorectal ultrasound, multidetector computed tomography (MDCT), and magnetic resonance imaging (MRI). The 7th TNM staging system was used to evaluate the primary tumor and lymph nodes. The measure of lymph node diameter was based on preoperative imaging. The treatment strategy was determined by the multidisciplinary team meeting (MDT). Patients with advanced rectal cancer (cT3–4 and/or cN+) received neoadjuvant chemoradiotherapy (nCRT) that consisted of concurrent capecitabine and radiotherapy at a total dose of 45 Gy or preoperative short-course radiotherapy with a total dose of 25 Gy. The operation was performed 8–12 weeks after the completion of the preoperative nCRT or within 1 week after preoperative short-course radiotherapy. For patients who had pathological stage III or stage II disease with a high risk of recurrence, 5-fluorouracil-based adjuvant chemotherapy was recommended.

### Surgical technique

**Fig. 2:**
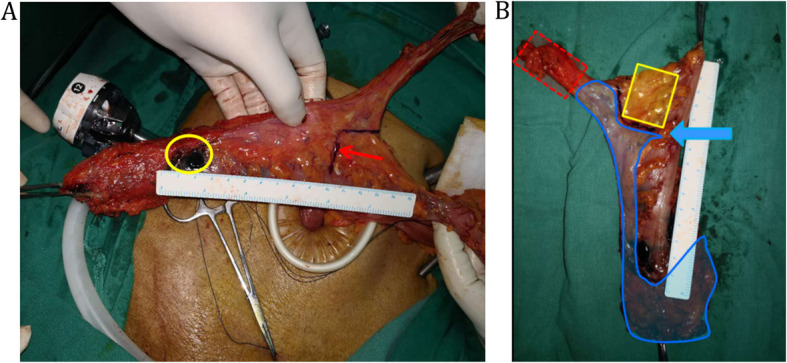
**a** After the distal bowel transection, the tumor-bearing bowel was pulled out of the abdominal cavity. The carbon nanoparticle suspension was injected into the subserosal layer around the tumor to trace lymph nodes (yellow circle). The point at 10 cm proximal to the primary tumor on the bowel wall was marked (red arrow); **b** Lymph nodes were mapping and harvested on the fresh specimen. In this study, mesenteric lymph nodes were classified into three parts: main lymph nodes lied along the IMA from the origin of LCA to the root of IMA (MLNs) (red area); superior rectal and perirectal lymph nodes (SPLNs) (blue area); pericolic lymph nodes located beyond 10 cm proximal to the tumor (PCNs) (yellow area). The blue arrow represented the level of 10 cm proximal to the primary tumor on the bowel wall

In this study, the standard total mesorectum excision (TME) procedure was performed according to our previously reported method [13]. Briefly, the mesorectum was sharply dissected along the Toldt’s space to preserve the plane integrity. Patients with a tumor located below peritoneal reflection underwent total mesorectum excision. For higher rectal cancer, the mesorectum resection margin of ≥5 cm and the distal resection margin of ≥3 cm were required. All patients received either high ligation of the inferior mesenteric artery (IMA) or main lymph node dissection with left colic artery (LCA) preservation. Patients with suspected lateral pelvic lymph nodes received lateral lymph nodes dissection. A larger extent of colon mobilization or splenic flexure mobilization was performed to acquire adequate bowel length, whenever a tension on the anastomosis was anticipated. Then, the proximal bowel was transected at the level of more than 10 cm above the lesion (Fig. 2). After removal of the tumor-bearing segment, bowel anastomosis or enterostomy was completed.

### Specimen pathological assessment

In this study, the measurement of bowel and lymph nodes retrieval was done in the operation room. After transection of the distal bowel and extraction of the tumor-bearing segment through a sub-umbilical mini-laparotomy, the point of 10 cm proximal to tumor was marked with either a sterilized marking pen or a clip and the proximal resection margin distance was measured before the proximal bowel transection (Fig. 2a). Then, the carbon nanoparticle suspension (Chongqing LUMMY Pharmaceutical Co., Chongqing, China) was injected into the subserosal layer at several points around the tumor to trace lymph nodes [14]. In this study, we classified the mesenteric lymph nodes into three parts: main lymph nodes lied along the IMA from the origin of LCA to the root of IMA (MLNs); superior rectal and perirectal lymph nodes (SPLNs); pericolic lymph nodes located beyond 10 cm proximal to the tumor (PCNs) (Fig. 2b). After the tumor-bearing bowel was removed, the trained surgeons cooperated with the pathologists (Jiang D and He D) immediately identified the three lymph nodes regions and isolated those lymph nodes on the fresh specimen and recorded their number and distribution. To preserve the intactness of the mesorectum around the primary tumor for evaluation of circumferential resection margin, the retrieval of SPLNs stopped at the level 3 cm proximal to the superior edge of the tumor. Perirectal nodes distal to that level were counted and evaluated by pathologists (Jiang D and He D) under the guideline suggested by the Association of Coloproctology of Great Britain & Ireland [15]. The final number of lymph nodes was counted by the pathologists (Jiang D and He D) after H&E staining of all “nodes” picked out by both surgeons and pathologists. The tumor pathological staging was according to the 7th AJCC Staging Manual.

#### Outcomes measure

The primary outcome of this study was to investigate the real incidence of pPCN. The second outcomes were to explore the risk factors for pPCN and to determine its prognostic significance in rectal cancer.

#### Follow-up

After operation, patients were followed up according to the NCCN guideline. As we previously described, all patients were followed up every 3 months for the first 2 years and then annually thereafter until 5 years [13]. Laboratory examinations including CEA and CA19–9 was performed every 3 months. Chest and abdominal MDCT scans were performed every 6 months for 2 years and henceforth annually. One colonoscopy examination would be performed 1 year after operation and repeated every 3 years if no lesions were confirmed.

### Statistical analyses

Statistical analysis was performed by using SPSS software version 20.0 (IBM Inc., Armonk, NY). Continuous variables were expressed by the median, minimum, and maximum values. Comparisons between two groups were made using t-test, Wilcoxon rank-sum test, χ^2^ test, or Fisher exact test. Kaplan-Meier method with the log-rank test was used to calculate the overall survival (OS) and disease-free survival (DFS). *P* values were derived from two-tailed tests and *p* value < 0.05 was considered statistically significant. Variables with a P value < 0.2 in univariate analysis were further evaluated in a multivariate analysis using Logistic regression analysis to identify independent risk factors for pPCN. The Cox proportional hazard regression model was used to assess the prognostic value of individual variables.

## Results

### Demographic characteristics and perioperative outcomes of the overall study population

A total of 312 consecutive patients with rectal cancer were included in this study. The demographic characteristics were shown in Table 1. The median age was 60 years. The male to female was 199:113. The median distance from the tumor to the anal verge was 6 cm. Overall, 32 % of patients had received preoperative nCRT. Of 312 rectal resection, 282 (90.4%) patients underwent laparoscopic surgery and 26 (8.3%) patients underwent open surgery. Two hundred and nine out of the 312 patients (67%) underwent low anterior resection. The median operation time and blood loss were 180 (range, 80–540) min and 40 (range, 5–450) ml, respectively. The median postoperative hospital stay was 8 (range, 3–48) days, and the median time to tolerance to liquid food was 72 (range, 10–432) hours. After operation, a total of 138 (44.2%) patients received adjuvant chemotherapy and 24 (7.7%) received adjuvant chemoradiotherapy. Sixty-five (20.8%) patients suffered various severity of postoperative complications.

**Table 1:** Clinicopathological and Preoperative Imaging Characteristics of Patients with Pericolic Lymph Nodes Metastasis Beyond 10 cm Proximal to The Tumor and Those without Metastasis on CT Scan

Variables	All patients (*n* = 312)	pPCN group (*n* = 14)	nPCN group (*n* = 298)	
	Value			*P* value
Age (year) ^a^	60 (27–94)	55.5 (44–79)	61 (27–94)	0.356
Sex				0.239
Male	199 (63.8%)	11 (78.6%)	188 (63.1%)	
Female	113 (36.2%)	3 (21.4%)	110 (36.9%)	
BMI (kg/m^2^)^a^	23.23 (15.07–32.33)	23.54 (17.72–27.68)	23.15 (15.07–32.33)	0.95
CEA (ng/ml)^a^	3.41 (0–1000)	4.34 (0.97–258.40)	3.41 (0–1000)	0.721
Distance from anal verge (cm) ^a^	6 (1–12)	7 (3–12)	6 (1–12)	0.766
Preoperative neoadjuvant chemoradiotherapy				**0.028**
Radiotherapy	31 (9.9%)	4 (28.6%)	27 (9.1%)	
Chemotherapy	15 (4.8%)	2 (14.3%)	13 (4.4%)	
Chemoradiotherapy	54 (17.3%)	1 (7.1%)	53 (17.8%)	
Operation procedure				1.00
Laparoscopy	282 (90.4%)	13 (92.9%)	269 (90.3%)	
Open	26 (8.3%)	1 (7.1%)	25 (8.4%)	
Type of surgery				0.331
Dixon	209 (67%)	9 (64.3%)	200 (67.1%)	
ELAPE or Miles	51 (16.3%)	4 (28.6%)	47 (15.7%)	
Hartmann	16 (5.1%)	0 (0%)	16 (5.4%)	
ISR	36 (11.5%)	1 (7.1%)	35 (11.7%)	
Operation time (min)^a^	180 (80–540)	177.5 (120–350)	180 (80–540)	0.459
Blood Loss (ml)^a^	40 (5–450)	40 (20–150)	40 (5–450)	0.736
Postoperative hospital stay (days)^a^	8 (3–48)	8 (5–20)	8 (3–48)	0.946
Time to tolerance to liquid food (hours)^a^	72 (10–432)	84 (24–432)	72 (10–336)	0.399
Postoperative complications	65 (20.8%)	2 (14.3%)	63 (21.1%)	0.779
Postoperative adjuvant chemoradiotherapy				**0.001**
Chemotherapy	138 (44.2%)	8 (57.1%)	130 (43.6%)	
Chemoradiotherapy	24 (7.7%)	5 (35.7%)	19 (6.4%)	
No. of mesenteric lymph nodes^a^	4.0 (0–20)	3.5 (2.0–14.0)	4.0 (0–20)	0.238
The maximum short-axis diameter of the largest mesenteric lymph node (mm)^a^	5.5 (0–19.0	7.4 (0–15.1)	5.5 (0–19.0)	**0.008**
Presence of mesenteric lymph nodes with the maximum short-axis diameter ≥ 8 mm	56 (17.9%)	7 (50%)	49 (16.4%)	**0.005**
Clinical T stage n (%)^b^				**0.049**
T1–2	60 (19.2%)	0 (0.0%)	60 (20.1%)	
T3–4	252 (80.8%)	14 (100%)	238 (79.9%)	
Clinical N stage n (%)^b^				**0.017**
N0	149 (47.8%)	3 (21.4%)	146 (49.0%)	
N1	124 (39.7%)	5 (35.7%)	119 (39.9%)	
N2	39 (12.5%)	6 (42.9%)	33 (11.1%)	
Clinical M stage n (%)				0.083
M0	288 (92.3%)	11 (78.6%)	277 (92.9%)	
M1	24 (7.7%)	3 (21.4%)	21 (7.1%)	
AJCC stage n (%) ^c^				**0.018**
I	50 (16%)	0 (0.0%)	50 (16.8%)	
II	94 (30.1%)	3 (21.4%)	91 (30.5%)	
III	144 (46.2%)	8 (57.2%)	136 (45.6%)	
IV	24 (7.7%)	3 (21.4%)	21 (7.1%)	

pPCN group, patients in this group with pericolic lymph nodes metastasis beyond 10 cm proximal to the tumor;

nPCN group, patients in this group without pericolic lymph nodes metastasis beyond 10 cm proximal to the tumor

Data were presented as n (%); ^a^Median (range); *BMI* body mass index, *CEA* carcinoembryonic antigen

^b^ Evaluation of T stage counted mainly on MRI; evaluation of N staging mainly on CT and MRI combined

^c^TNM stage was classified according to American Joint Committee on Cancer (AJCC)

### Clinicopathological and preoperative imaging characteristics between the pPCN group and nPCN group

Based on the pathological findings, 14 patients (4.5%) with pPCN were confirmed (Table 1). The clinicopathological characteristics were compared between the pPCN group and nPCN group (Table 1). Basic demographic characteristics and perioperative outcomes were balanced except that more patients in pPCN group received preoperative nCRT and postoperative adjuvant therapy. Preoperative imaging characteristics on CT and MRI scans were also shown in Table 1. The median maximum short-axis diameter of the largest mesenteric lymph node was larger in pPCN group than in nPCN group (7.4 mm vs 5.5 mm, *p* = 0.008). The percent of patients with the maximum short-axis diameter of mesenteric lymph node ≥8 mm was higher in pPCN group than in nPCN group (50% vs 16.4%, *p* = 0.005). There were significantly more advanced clinical T stage, N stage, and TNM stage distribution in pPCN group than in nPCN group. More patients in pPCN group had cT3–4 stage or cN2 stage than in nPCN group (100% vs 79.9%, *p* = 0.049; 42.9% vs 11.1%, *p* = 0.017, respectively). Seventy-eight percent of patients had cTNM stage III-IV disease in pPCN group compared with 52.7% of patients in nPCN group.

Table 2 showed the pathological characteristics between the pPCN group and nPCN group. The maximal diameter of tumor between the two groups was similar. No significant difference was found in the median number of retrieved lymph nodes, yet the median number of positive mesenteric lymph nodes was more in pPCN group than in nPCN group (3.5 vs 0, *p* = 0.002). Furthermore, patients in pPCN group had more PCNs (4.5 vs 0, *p* <  0.001). In this study, more than half of patients (51.6%) had obtained a median of 3 PCNs. The number of SPLNs, MLNs, and positive MLNs were no significant differences between the groups. However, compared with nPCN group, the median number of positive SPLNs was more in pPCN group (2 vs 0, *p* = 0.007). Similar to the clinical TNM stage, a higher percentage of advanced pathological T3–4 stage (92.9% vs 55.4%), N2 stage (50% vs 5.7%), and III-IV stage (100% vs 34.2%) were observed in the pPCN group. Additionally, poor grading (G3/G4) and vascular invasion were also more common in pPCN group compared with nPCN group (71.4% vs 23.2%, *p* = 0.001; 35.7% vs 8.7%, *p* = 0.007, respectively).

**Table 2:** Pathological Characteristics of Patients with Pericolic Lymph Nodes Metastasis Beyond 10 cm Proximal to The Tumor and Those without Metastasis

Variables	All patients (*n* = 312)	pPCN group (*n* = 14)	nPCN group (*n* = 298)	
	Value			*P* value
Maximum size (cm)^a^	3.2 (0–8.0)	3.75 (2.0–6.0)	3.0 (0–8.0)	0.153
Total no. of mesenteric lymph nodes harvested^ab^	14 (0–45.0)	17.5 (5.0–38.0)	13.5 (0–45.0)	0.218
Total no. of positive mesenteric lymph nodes^ab^	0 (0–11.0)	3.5 (1.0–14.0)	0 (0–11.0)	**0.002**
No. of PCNs^a^	1 (0–12.0)	4.5 (1.0–11.0)	0 (0–12.0)	**< 0.001**
No. of positive PCNs^a^	0 (0–6.0)	1 (1.0–6.0)	0	**< 0.001**
Patients with PCNs	161 (51.6%)	14 (100%)	147 (49.3%)	**< 0.001**
No. of SPLNs^a^	6 (0–26.0)	9.0 (0–13.0)	6.0 (0–26.0)	0.099
No. of positive SPLNs^a^	0 (0–9.0)	2.0 (0–9.0)	0 (0–9.0)	**0.007**
No. of MLNs^a^	1.0 (0–19.0)	2.0 (0–17.0)	1.0 (0–19.0)	0.153
No. of positive MLNs^a^	0 (0–3.0)	0 (0–3.0)	0 (0–3.0)	0.206
pT stage				**0.006**
T0–2	134 (42.9%)	1 (7.1%)	133 (44.6%)	
T3–4	178 (57.1%)	13 (92.9%)	165 (55.4%)	
pN stage				**< 0.001**
N0	205 (65.7%)	0 (0%)	205 (68.8%)	
N1	83 (26.6%)	7 (50%)	76 (25.5%)	
N2	24 (7.7%)	7 (50%)	17 (5.7%)	
pTNM stage				**< 0.001**
0-II	196 (62.8%)	0 (0%)	196 (65.8%)	
III-IV	116 (37.2%)	14 (100%)	102 (34.2%)	
Histological type				**0.001**
G1/G2	211 (67.6%)	4 (28.6%)	207 (69.5%)	
G3/G4	79 (25.3%)	10 (71.4%)	69 (23.2%)	
Cancer nodule	41 (13.1%)	4 (28.6%)	37 (12.4%)	0.096
Vascular invasion	31 (9.9%)	5 (35.7%)	26 (8.7%)	**0.007**
Nerve invasion	61 (19.6%)	4 (28.6%)	57 (19.1%)	0.487
Positive circumferential resection margin	10 (3.2%)	2 (14.3%)	8 (2.7%)	0.140

pPCN group, patients in this group with pericolic lymph nodes metastasis beyond 10 cm proximal to the tumor

nPCN group, patients in this group without pericolic lymph nodes metastasis beyond 10 cm proximal to the tumor

Data were presented as n (%); ^a^Median (range)

^b^Mesenteric lymph nodes including PCNs,SPLNs, and MLNs

PCNs,pericolic lymph nodes located beyond 10 cm proximal to the tumor

SPLNs, superior rectal and perirectal lymph nodes

MLNs, main lymph nodes lied along the inferior mesenteric artery (IMA) from the origin of the left colic artery (LCA) to the root of IMA

### Risk factors for pPCN

The univariate analysis of risk factors for pPCN revealed that preoperative imaging characteristics including the maximum short-axis diameter of the largest mesenteric lymph node, mesenteric lymph nodes of ≥8 mm, and advanced cTNM stage were significantly associated with pPCN. Additionally, more PCNs retrieval, more positive mesenteric lymph nodes, more positive SPLNs, more advanced pTNM stage, more vascular invasion, and worse grading were also among risk factors for pPCN. After multivariate analysis, postoperative pathological variables including number of PCNs retrieval and total number of positive mesenteric lymph nodes were independent risk factors for pPCN (OR 5.156, 95% CI 2.152–12.356, *P* <  0.05; OR 1.868, 95% CI 1.257–2.775, *P* = 0.002, respectively) (Table 3). However, only cTNM remained a preoperative independent risk factor for predicting pPCN (OR 11.749, 95% CI 2.121–65.081, *P* = 0.005) (Table 3). Although without statistical significance, the presence of the largest mesenteric lymph nodes with the maximum short-axis diameter ≥ 8 mm on imaging had the potential to become an independent predicting indicator (OR 5.571, 95% CI 0.839–37.0, *P* = 0.075) in a larger series.

**Table 3:** Multivariate Analysis of Clinicopathological Features Associated with Pericolic Lymph Nodes Metastasis beyond 10 cm Proximal to The Tumor

Variables	Univariate analysis			Multivariate analysis		
	pPCN group (*n* = 14)	nPCN group (*n* = 298)	*P* value	OR	95% CI	*P* value
cTNM stage n(%)			0.018	11.749	2.121–65.081	0.005
I	0 (0.0%)	50 (16.8%)				
II	3 (21.4%)	91 (30.5%)				
III	8 (57.2%)	135 (45.3%)				
IV	3 (21.4%)	22 (7.4%)				
No. of PCNs	4.5 (1.0–11.0)	0 (0–12.0)	< 0.001	1.868	1.257–2.775	0.002
Total no. of positive mesenteric lymph nodes	3.5 (1.0–14.0)	0 (0–11.0)	0.002	5.156	2.152–12.356	< 0.05
Presence of the largest mesenteric lymph nodes with the maximum short-axis diameter ≥ 8 mm	7 (50%)	49 (16.4%)	0.005	5.571	0.839–37.0	0.075

pPCN group, patients in this group with pericolic lymph nodes metastasis beyond 10 cm proximal to the tumor

nPCN group, patients in this group without pericolic lymph nodes metastasis beyond 10 cm proximal to the tumor

PCNs, pericolic lymph nodes located beyond 10 cm proximal to the tumor

*OR* odds ratio, *CI* confidence interval

### Medium-term outcomes of patients between the pPCN group and nPCN group

**Fig. 3:**
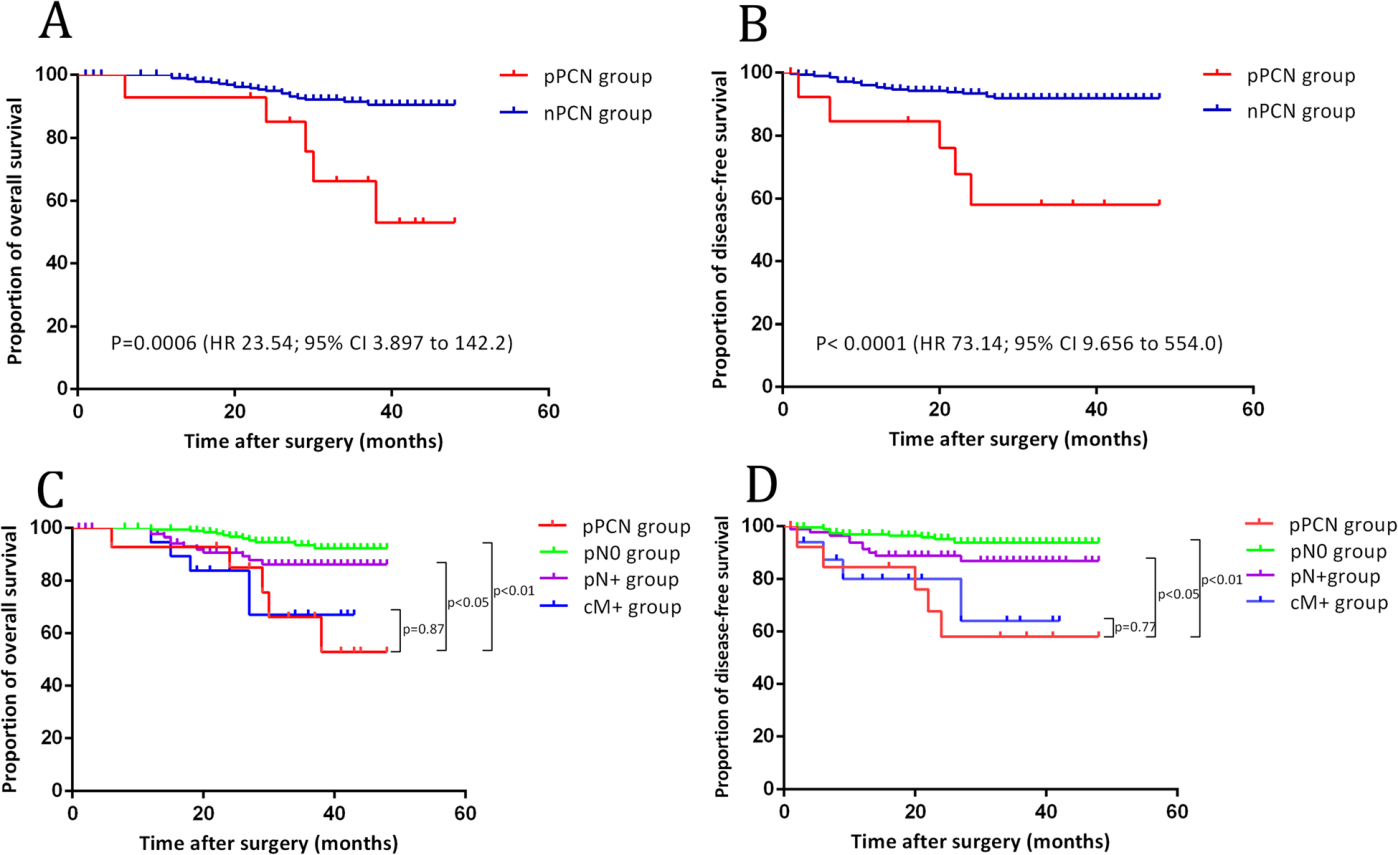
**a** The 3-year cumulative disease-free survival (DFS) was compared in patients with pPCN and patients with nPCN; **b** The 3-year cumulative overall survival (OS) was compared between patients with pPCN and patients with nPCN; **c** The 3-year OS was compared between patients with pPCN and other patients with advanced N stage or cM stage; **d** The 3-year DFS was compared between patients with pPCN and other patients with advanced N stage or cM stage

During the follow-up period, follow-up data were available for 309 (99%) patients and only three (1%) patients were lost to follow-up. The median follow-up duration in the overall study population was 32 months (range from 0 to 48). No local recurrence was developed in patients with pPCN at the end of follow-up. For the whole patients, the 3-year OS and DFS were 90.1, 90.3% respectively. The 3-year OS was 91% in patients with nPCN and 66% in patients with pPCN (hazard ratios (HR) 23.54, 95% CI 3.897 to 142.2, *p* = 0.0006) (Fig. 3a). The 3-year DFS was 92% in patients with nPCN and 58% in patients with pPCN (HR 73.14, 95% CI 9.656 to 554.0, *p* <  0.0001) (Fig. 3b).

Furthermore, the nPCN group was divided into pN0, pN(+), and cM1 group to investigate whether positive PCNs presented different prognostic significance. The 3-year OS and DFS were significantly worse in patients with positive PCNs than in other patients with positive MLNs or SPLNs (HR 4.37, 95% CI 1.052 to 18.17, *p* = 0.042; HR 6.59, 95% CI 1.431 to 30.33, *p* = 0.016, respectively) (Fig. 3c, d). The Cox proportional hazards regression model suggested that pPCN was an independent indicator for poor 3-year OS and DFS (HR 4.433, 95% CI 1.124 to 17.485, *p* = 0.033; HR 6.703, 95% CI 1.508 to 29.795, *P* = 0.012, respectively) (Table 4). Additionally, we performed a subgroup analysis to identify the independent prognostic factors in those patients with stage III-IV. We still found that pPCN was an independent prognostic factor for DFS. Due to the limit of sample size and statistical test efficiency, there was still a trend toward that pPCN was an independent prognostic factor for OS (supplement Table 2).

**Table 4:** Univariate and multivariate Cox regression analysis of prognostic factors for overall survival and disease-free survival in patients with rectal cancer

Variable	Overall survival				Disease-free survival			
	Univariate HR (95% CI)	*p* value	Multivariate HR (95% CI)	*p* value	Univariate HR (95% CI)	*p* value	Multivariate HR (95% CI)	*p* value
Age (< 60/≥60 y)	1.196 (0.560–2.554)	0.645	2.411 (0.953–6.102)	0.063	1.152 (0.539–2.461)	0.715	2.444 (0.960–6.220)	0.061
Gender (Male/Female)	0.574 (0.242–1.356)	0.206	0.766 (0.295–1.988)	0.583	0.572 (0.242–1.352)	0.203	0.759 (0.295–1.949)	0.566
CEA (< 5/≥5 ng/ml)	1.999 (0.944–4.232)	0.07	1.054 (0.438–2.536)	0.907	**2.148 (1.013–4.553)**	**0.046**	1.186 (0.492–2.859)	0.704
Distance from anal verge (< 6/≥6 cm) *	0.687 (0.323–1.461)	0.329	0.493 (0.190–1.283)	0.147	0.657 (0.309–1.398)	0.275	0.428 (0.165–1.108)	0.08
Neoadjuvant chemoradiotherapy (No/Yes)	**2.939 (1.375–6.281)**	**0.005**	**3.119 (1.256–7.744)**	**0.014**	**3.096 (1.447–6.620)**	**0.004**	**3.604 (1.450–8.958)**	**0.006**
Maximum size (< 3/≥3 cm)*	1.506 (0.637–3.562)	0.351	1.448 (0.521–4.026)	0.478	1.532 (0.648–3.624)	0.331	1.511 (0.507–4.508)	0.459
No. of total mesenteric lymph nodes harvested (< 12/≥12)	1.350 (0.570–3.200)	0.495	1.726 (0.509–5.855)	0.381	1.494 (0.632–3.534)	0.361	1.898 (0.572–6.305)	0.295
No. of PCNs (< 2/≥2)	1.124 (0.524–2.414)	0.764	0.681 (0.227–2.048)	0.494	1.239 (0.582–2.637)	0.578	0.580 (0.178–1.897)	0.368
No. of SPLNs (< 6/≥6)	0.928 (0.432–1.993)	0.847	0.606 (0.211–1.739)	0.352	1.039 (0.486–2.221)	0.921	0.557 (0.191–1.623)	0.283
No. of MLNs (< 2/≥2)	1.410 (0.648–3.071)	0.387	2.304 (0.867–6.122)	0.094	1.597 (0.741–3.442)	0.232	2.317 (0.865–6.207)	0.095
Pericolic lymph nodes metastasis beyond 10 cm proximal to the tumor (No/Yes)	**v4.689 (1.775–12.386)**	**0.002**	**4.433 (1.124–17.485)**	**0.033**	**5.799 (2.190–15.352)**	**< 0.001**	**6.703 (1.508–29.795)**	**0.012**
pT stage (T0–2/T3–4)	2.346 (0.555–9.917)	0.246	2.808 (0.543–14.534)	0.218	2.401 (0.568–10.142)	0.234	2.694 (0.516–14.072)	0.24
pN stage (N0/N1–2)	**2.940 (1.364–6.335)**	**0.006**	2.033 (0.918–4.504)	0.08	**3.294 (1.527–7.103)**	**0.002**	**2.606 (1.084–6.266)**	**0.032**
pTNM stage (0-II/III-IV)	**4.418 (1.934–10.094)**	**< 0.001**	**12.053 (2.782–52.223)**	**0.001**	**5.129 (2.242–11.733)**	**< 0.001**	**21.327 (4.322–105.224)**	**< 0.001**
Histological type (G1–2/G3–4)	1.104 (0.481–2.533)	0.815	0.608 (0.209–1.763)	0.359	1.238 (0.542–2.829)	0.612	0.682 (0.229–2.033)	0.492
Cancer nodule (No/Yes)	**2.589 (1.094–6.124)**	**0.03**	1.494 (0.436–5.127)	0.523	**2.963 (1.250–7.022)**	**0.014**	1.703 (0.495–5.859)	0.398
Vascular invasion (No/Yes)	1.312 (0.395–4.364)	0.657	0.541 (0.142–2.063)	0.368	1.351 (0.407–4.486)	0.624	0.653 (0.177–2.406)	0.522
Nerve invasion (No/Yes)	1.702 (0.717–4.039)	0.228	1.151 (0.434–3.055)	0.777	1.667 (0.705–3.944)	0.245	1.154 (0.441–3.017)	0.77
Circumferential resection margin (Negative/Positive)	3.013 (0.712–12.753)	0.134	2.049 (0.379–11.064)	0.404	2.734 (0.646–11.569)	0.172	0.781 (0.134–4.568)	0.784

PCNs, pericolic lymph nodes located beyond 10 cm proximal to the tumor

SPLNs, superior rectal and perirectal lymph nodes

MLNs, main lymph nodes lied along the inferior mesenteric artery (IMA) from the origin of the left colic artery (LCA) to the root of IMA

*HR* hazard ratio, *CI* confidence interval

## Discussion

According to our findings, 4.5% of patients with pPCN were confirmed. To analyze the status of proximal pericolic lymph nodes in colorectal cancer, we systematically searched relevant literature and made a narrative synthesis (supplement Table 2). We found that the incidence of pPCN in this study was higher compared to previous studies with the incidence ranged from 0 to 1.8% [8–11]. In those studies, however, the distance from the primary tumor to the proximal resection margin was measured ex vivo condition after proximal bowel transection or lymph nodes were retrieved on specimens fixed with formalin. As we know, the shrinkage of the length of bowel and its mesentery was highly variable after bowel transection and fixation. Bhatnagar et al. reported the shrinkage of sigmoid colon after fixation was about 25–40%, which mainly occurred in the sigmoid mesocolon [16]. Goldstein et al. reported that the bowel segments shrank 57% of the in vivo length, among which 70% of the shrinkage occurred during the first 10–20 min after removal and 30% occurred after fixation [17]. One recent study also demonstrated that 10–20% shrinkage or 1 mm size reduction occurred in lymph nodes after formalin fixation [18]. However, different from previous reports, in this study, the margin distance was measured in vivo condition, the area to harvest PCNs was marked out in operation, and lymph nodes were harvested immediately on the fresh specimens. The avoidance of ex vivo specimen shrinkage might have contributed to the higher incidence of positive PCNs observed in this study. In this regard, this study pointed out, for the first time, that surgeons should be careful to apply the former seemingly “well-established” 5 or 10 cm rule in surgery for rectal cancer. This was especially true for patients with more advanced disease (cTNM stage III-IV) who had an even higher rate of positive PCNs (6.5%).

Due to ideal effectiveness and few side effects, carbon nanoparticle was used to trace lymph nodes in different cancers such as breast cancer, thyroid cancer, and colorectal cancer [14]. One recent meta-analysis demonstrated that carbon nanoparticle labeling lymph nodes could improve the retrieved number of lymph nodes in colorectal resection [19]. In the present study, we also attempted to use the nano-carbon tracer method to trace lymph nodes. After the tumor-bearing bowel resection, the trained surgeons and pathologists immediately retrieved lymph nodes from fresh specimens and grouped them based on the above classification. Based on our methods, a median number of 14 lymph nodes in the overall population, 17.5 lymph nodes in the pPCN group, and 13.5 in nPCN group were harvested. Additionally, overall, 51.6% of patients with a median number of 3 PCNs were observed. As the AJCC recommends, it is necessary to obtain an adequate number of lymph nodes (≥ 12) for accurate staging and identifying patients who need postoperative chemoradiotherapy [20]. Nowadays, preoperative radiotherapy followed by curative resection has become the standard treatment for locally advanced rectal cancer. However, preoperative radiotherapy decreases the number of analyzable lymph nodes [21]. In our study, although 32 % of patients received preoperative nCRT, an adequate number of lymph nodes were obtained, which was contributed to validating appropriate staging.

Overall, there was limited evidence involving upward pericolic lymph nodes metastasis in patients with rectal cancer. In the present study, the incidence of pPCN (4.5%) was higher than expected and these regional lymph nodes (PCNs) metastasis were worthy of further evaluation. When extended bowel resection was performed, a larger extent of colon mobilization was required to ensure a tension-free anastomosis. Therefore, for patients with pPCN, extended proximal bowel resection with splenic flexure mobilization was required to remove these positive PCNs and obtain tension-free anastomosis. However, colorectal surgeons in the east do not perform routine splenic flexure mobilization in low anterior resection in rectal cancer [22]. Thus, before operation, it was necessary to identify high-risk factors to predict patients with pPCN. Based on the univariate and multivariate analysis, preoperative and intraoperative characteristics including more advanced cTNM stage (III-IV), the larger short-axis diameter of the largest mesenteric lymph node, the presence of mesenteric lymph node with the maximum short-axis diameter ≥ 8 mm, and more PCNs were contributed to identifying those patients with high-risk local recurrence. Therefore, when surgery was performed for those with unfavorable biological features, the possibility for potential pPCN should be kept in mind. In the present study, no local recurrence was developed in patients with pPCN after extended proximal resection with removing the positive PCNs. Therefore, based on our experience, for patients with preoperative suspected pPCN, splenic flexure mobilization with extended bowel resection was recommended to obtain adequate oncological resection and avoid residual positive PCNs. Moreover, a colonic J-pouch is recommended as a reasonable approach to improving functional outcomes after a low anterior resection for rectal cancer [23]. If the 4.5% of patients with pPCN were performed with sigmoid colonic J-pouch procedure, they may have a risk of local recurrence for no adequate pericolic lymph nodes dissection which was based on the 5-cm or 10-cm rule. Additionally, as 32% of patients received preoperative neoadjuvant chemoradiotherapy in this study, some studies found that radiation-induced injury existed in bowel resection margin after preoperative radiotherapy [24]. Thus, for those with preoperative radiotherapy underwent extended proximal resection could potentially reduce the risk of anastomosis complication [25].

Some previous studies concerned the relationship between the proximal bowel resection length and oncological outcomes in patients with colorectal cancer (supplement Table 3) [5, 6, 26–28]. Overall, survival outcomes were worse in patients with proximal bowel resection margin less than 5 cm than that in those with proximal bowel resection margin more than 5 cm. However, few studies explored the relationship between the proximal resection margin more than 10 cm and long-term oncological outcomes in rectal cancer. In the present study, we further explored the oncological outcomes of patients with or without pPCN. We found that the 3-year OS and DFS of patients with pPCN were significantly worse than that of patients with nPCN. Furthermore, according to the subgroup analysis, compared to other patients with positive MLNs or SPLNs, patients with positive PCNs still had significantly worse 3-year OS and DFS which even were similar to that in patients with distant organ metastasis. pPCN accompanied by multiple high-risk factors including poor tumor differentiation, vascular invasion, and more positive mesenteric nodes might contribute to the worse survival. In this study, compared with nPCN group, although more patients in the pPCN group had received preoperative nCRT or postoperative chemoradiotherapy, more survival benefits were not obtained. Taking the results of COX analysis into consideration, we believed that pPCN was an independent poor prognostic risk.

There were some limitations in our studies. Firstly, although there was no significant difference in demographic characteristics between the pPCN group and nPCN group, the sample size in pPCN group did not suffice the identification of more preoperative risk factors for predicting pPCN. Secondly, the optimal proximal resection margin in rectal cancer still cannot be confirmed in this study. Before the commencement of this study, in the preliminary phase, we tried to collect paracolic lymph nodes of 10–15 cm from the tumor and those beyond 15 cm separately. However, resection of up to 15–20 cm proximal bowel often warrants the full mobilization of the splenic flexure, which is far from a routine maneuver in eastern Asian countries. Furthermore, the harvest of lymph nodes was often zero. Hence, we abandoned this. Even when a proximal resection of greater than 15 cm was achieved, we collected the lymph nodes in only one group (PCNs). Thus, we commenced this study with the main aim to investigate the incidence of pPCN and its prognostic value, rather than to confirm the optimal proximal resection margin in rectal cancer. Thirdly, our results revealed the pPCN as an independent poor prognostic factor. The survival curve of pPCN patients fell in the same range as that of stage IV patients. It seemed like an extended proximal resection margin might be valueless for those patients with pPCN. Due to the limited cases with pPCN, a similar prognosis between pPCN and stage IV patients did not represent that they had the same results. Furthermore, as stage IV patients were curable, we believed that patients with pPCN were also heterogeneous in prognosis. Removal of longer bowel might avoid residual positive PCNs which would very likely lead to local recurrence if those patients did not die of distant metastasis first. Additionally, for stage IV patients, it is important to prevent perioperative complications and improve the quality of life. Thus, an extended proximal bowel resection should be a caution to perform in these patients. Lastly, a small-size sample in nPCN group was not allowed us to perform a matched analysis. More prospective studies with a large sample size were needed.

## Conclusions

Our study indicated that the real incidence of pPCN which was based on intraoperative measurement was higher than expected and patients with pPCN had worse oncological outcomes. Our results suggested that there was a possible advantage of extended bowel resection with the proximal resection margin more than 10 cm in patients with more advanced disease (cTNM stage III) or more PCNs found in operation, which contribute to avoiding residual positive PCNs. The optimal length of proximal bowel resection, as well as the impact of longer bowel resection on outcomes, remains to be clarified in larger prospective cohorts.

## Supplementary information

**Additional file 1: Supplement Table 1**. Cox regression analysis of prognostic factors for overall survival and disease-free survival in patients with IV stage rectal cancer.

**Additional file 2: Supplemental Table 2**. Literature concerned on the incidence of proximal pericolic lymph nodes metastasis from colorectal cancer.

**Additional file 3: Supplemental Table 3**. Literature concerned the relationship between the length of proximal bowel resection and oncological outcomes in patients with colorectal cancer.

## Data Availability

The datasets used and/or analyzed during the current study are available from the corresponding author on reasonable request.

## Abbreviations

pPCN: Pericolic lymph nodes metastasis beyond 10 cm proximal to the tumor
nPCN: No pericolic lymph nodes metastasis beyond 10 cm proximal to the tumor
OS: Overall survival
DFS: Disease-free survival
nCRT: Neoadjuvant chemoradiotherapy
TME: Total mesorectum excision
IMA: Inferior mesenteric artery
LCA: Left colic artery
MLNs: Main lymph nodes lied along the IMA from the origin of LCA to the root of IMA
SPLNs: Superior rectal and perirectal lymph nodes
PCNs: Pericolic lymph nodes beyond 10 cm proximal to the tumor
