# Supplementary material for: The effect of pericolic lymph nodes metastasis beyond 10 cm proximal to the tumor on patients with rectal cancer
Source: BMC Cancer. 2020 Jun 19;20:573. doi: 10.1186/s12885-020-07037-3 (PMC7304140; doi:10.1186/s12885-020-07037-3)
Supplement: Supplementary file 1 — Additional file 1: Supplement Table 1. Cox regression analysis of prognostic factors for overall survival and disease-free survival in patients with IV stage rectal cancer. [file 12885_2020_7037_MOESM1_ESM.doc]

| **Supplement Table1. Cox regression analysis of prognostic factors for overall survival and disease-free survival in patients with IV stage rectal cancer.** | | | | |
| --- | --- | --- | --- | --- |
| Variable | Overall survival | | Disease-free survival | |
|  | Multivariate HR (95% CI) | p value | Multivariate HR (95% CI) | p value |
| Age (<60/≥60 y) | 1.309 (0.41-4.177) | 0.649 | 1.262 (0.391-4.079) | 0.697 |
| Gender (Male/Female) | 1.315 (0.42-4.113) | 0.638 | 1.274 (0.415-3.91) | 0.672 |
| CEA (<5/≥5 ng/ml) | 1.395 (0.412-4.723) | 0.593 | 1.844 (0.55-6.182) | 0.321 |
| Distance from anal verge (<6/≥6 cm) * | 0.888 (0.235-3.353) | 0.861 | 0.78 (0.212-2.87) | 0.708 |
| Neoadjuvant chemoradiotherapy (No/Yes) | 1.81 (0.572-5.733) | 0.313 | 2.207 (0.703-6.932) | 0.175 |
| Maximum size (<3/≥3 cm)* | 1.296 (0.316-5.318) | 0.719 | 0.988 (0.221-4.409) | 0.987 |
| No. of total mesenteric lymph nodes harvested (<12/≥12) | 2.375 (0.425-13.287) | 0.325 | 2.555 (0.505-12.935) | 0.257 |
| No. of PCNs (<2/≥2) | 1.238 (0.326-4.693) | 0.754 | 0.92 (0.218-3.88) | 0.91 |
| No. of SPLNs (<6/≥6) | 0.278 (0.067-1.148) | 0.077 | 0.248 (0.06-1.017) | 0.053 |
| No. of MLNs (<2/≥2) | 1.621 (0.445-5.9) | 0.464 | 1.854 (0.502-6.842) | 0.354 |
| **Pericolic lymph nodes metastasis beyond 10 cm proximal to the tumor (No/Yes)** | **3.386 (0.829-13.829)** | **0.089** | **5.579 (1.216-25.604)** | **0.027** |
| pT stage (T0-2/T3-4) | 4.31 (0.479-38.796) | 0.193 | 6.163 (0.588-64.595) | 0.129 |
| pN stage (N0/N1-2) | 0.461 (0.083-2.55) | 0.375 | 0.389 (0.056-2.687) | 0.339 |
| Histological type (G1-2/G3-4) | 0.422 (0.117-1.518) | 0.186 | 0.429 (0.115-1.603) | 0.208 |
| Cancer nodule (No/Yes) | 1.133 (0.303-4.243) | 0.853 | 1.151 (0.313-4.227) | 0.832 |
| Vascular invasion (No/Yes) | 0.293 (0.054-1.599) | 0.156 | 0.356 (0.072-1.759) | 0.205 |
| Nerve invasion (No/Yes) | 1.358 (0.44-4.192) | 0.594 | 1.38 (0.461-4.132) | 0.565 |
| Circumferential resection margin (Negative/Positive) | 2.755 (0.436-17.388) | 0.281 | 1.071 (0.165-6.962) | 0.943 |
| PCNs, pericolic lymph nodes located beyond 10 cm proximal to the tumor; SPLNs, superior rectal and perirectal lymph nodes; MLNs, main lymph nodes lied along the inferior mesenteric artery (IMA) from the origin of the left colic artery (LCA) to the root of IMA; HR, hazard ratio; CI, confidence interval. | | | | |
