# Supplementary material for: The effect of pericolic lymph nodes metastasis beyond 10 cm proximal to the tumor on patients with rectal cancer
Source: BMC Cancer. 2020 Jun 19;20:573. doi: 10.1186/s12885-020-07037-3 (PMC7304140; doi:10.1186/s12885-020-07037-3)
Supplement: Supplementary file 2 — Additional file 2: Supplemental Table 2. Literature concerned on the incidence of proximal pericolic lymph nodes metastasis from colorectal cancer. [file 12885_2020_7037_MOESM2_ESM.doc]

| **Supplemental table 2. Literature concerned on the incidence of proximal pericolic lymph nodes metastasis from colorectal cancer.** | | | | | | |
| --- | --- | --- | --- | --- | --- | --- |
| Author | Year | No. of patients | The incidence of proximal pericolic lymph node metastasis | | | The length of proximal bowel resection (cm) |
|  |  |  | < 5cm | 5-10 cm | ≥ 10 cm |  |
| Morikawa E^8^ | 1994 | 171 | 25.7% | 8.2% | 1.8% | - |
| Kameda K^9^ | 1990 | 44 | 15.9% | 20.5% | 0 | - |
| Toyota S^10^ | 1995 | 328 | 7.3% | 0.6% | 0.3% | 10 |
| Hida J^11^ | 2005 | 164 | 20.1% | - | 0.6% | >10 |
