# Supplementary material for: The effect of pericolic lymph nodes metastasis beyond 10 cm proximal to the tumor on patients with rectal cancer
Source: BMC Cancer. 2020 Jun 19;20:573. doi: 10.1186/s12885-020-07037-3 (PMC7304140; doi:10.1186/s12885-020-07037-3)
Supplement: Supplementary file 3 — Additional file 3: Supplemental Table 3. Literature concerned the relationship between the length of proximal bowel resection and oncological outcomes in patients with colorectal cancer. [file 12885_2020_7037_MOESM3_ESM.doc]

| **Supplemental table 3. Literature concerned the relationship between the length of proximal bowel resection and oncological outcomes in patients with colorectal cancer.** | | | | | | | |
| --- | --- | --- | --- | --- | --- | --- | --- |
| Author | Year | No. of patients | OS/DFS/LR | | | The length of proximal bowel resection (cm) | Statistical significance* |
|  |  |  | < 5cm | 5-10 cm | ≥ 10 cm |  |  |
| Copeland EM^5^ | 1968 | 347 | 5-year OS 38.4% | 5-year OS 51.1% | | > 5 | Yes |
| Devereux DF^6^ | 1985 | 214 | Dukes' B 2-year LR 20% | Dukes' B 2-year LR 5% | | > 5 | Yes |
| Saha AK^26^ | 2011 | 180 | 5-year OS 48% | 5-year OS 65% | | > 5 | Yes |
| Rocha R^27^ | 2016 | 215 | LR 10.1% | 5-year LR 6.9% | | > 5 | No |
|  |  |  | time-to-recurrence 21.8 months | time-to-recurrence 32.3 months | |  | Yes |
|  |  |  | OS 52.9 months | OS 54.9 months | |  | No |
| [Lee SY](https://www.ncbi.nlm.nih.gov/pubmed/?term=Lee SY[Author]&cauthor=true&cauthor_uid=27996215)^28^ | 2017 | 1343 | 5-year OS 89% | 5-year OS 92.1% | 5-year OS 91.8% | > 10 | No |
| OS, overall survival; DFS, disease-free survival; LR, local recurrence;  *Statistical significance indicated that patients could get oncological advantage from extend bowel resection. | | | | | | | |
